# Discontinuing financial incentives for adherence to antipsychotic depot medication: long-term outcomes of a cluster randomised controlled trial

**DOI:** 10.1136/bmjopen-2016-011673

**Published:** 2016-09-21

**Authors:** Stefan Priebe, Stephen A Bremner, Hana Pavlickova

**Affiliations:** 1 Unit for Social and Community Psychiatry (WHO Collaborating Centre for Mental Health Service Development), Queen Mary University London, London, UK; 2 Division of Primary Care and Public Health, Brighton and Sussex Medical School, Brighton, UK

**Keywords:** Long-acting antipsychotic injectables (LAIs), financial incentives, psychosis, long-term effect

## Abstract

**Objectives:**

In a cluster randomised controlled trial, offering financial incentives improved adherence to antipsychotic depot medication over a 1-year period. Yet, it is unknown whether this positive effect is sustained once the incentives stop.

**Methods and analyses:**

Patients in the intervention and control group were followed up for 2 years after the intervention. Primary and secondary outcomes were assessed at 6 months and 24 months post intervention. Assessments were conducted between September 2011 and November 2014.

**Results:**

After the intervention period, intervention and control groups did not show any statistically significant differences in adherence, neither in the first 6 months (71% and 77%, respectively) nor in the following 18 months (68%, 74%). There were no statistically significant differences in secondary outcomes, that is, adherence ≥95% and untoward incidents either.

**Conclusions:**

It may be concluded that incentives to improve adherence to antipsychotic maintenance medication are effective only for as long as they are provided. Once they are stopped, adherence returns to approximately baseline level with no sustained benefit.

**Trial registration number:**

ISRCTN77769281; Results.

**Strengths and limitations of this study**

- Patients have been followed up for 24 months post intervention to examine shorter-term and longer-term effects.
- Data were not complete and likely not to be missing at random.
- A number of sensitivity analyses support the main results.

Poor adherence to antipsychotic maintenance medication is common in patients with psychotic disorders^1^ and linked to a number of negative outcomes.^2^ A range of interventions have been tested to improve adherence to antipsychotic medication, but hardly any has been shown to be effective. For example, so-called Compliance Therapy has explicitly not been recommended by the National Institute of Clinical Excellence. Thus, improving adherence in this patient group remains a challenge in treatment.

Against this background, clinicians in East London tried financial incentives to influence patients' adherence to long-acting injectable antipsychotics (LAIs). They reported positive outcomes in a small number of patients.^3^ A subsequent focus group study explored the concerns of different stakeholders about the idea of providing financial incentives to increase adherence to antipsychotic medication. While different concerns were raised, there was wide agreement that it would be important to go beyond anecdotal evidence and establish the effectiveness of financial incentives in a rigorous trial.^4^ This led to the design and funding of a cluster randomised controlled trial.^5^

The trial included patients with psychotic disorders treated in secondary mental health teams in the community in England and compared financial incentives with treatment as usual. The findings demonstrated that offering financial incentives of £15 (US$22, €17) per LAI over a 1-year period significantly improves adherence to LAIs.^6^ ^7^ Adherence already improved within the first 3 months of introducing incentives, and a significant difference with better adherence in patients being offered incentives was sustained over the full 1-year intervention period.^8^ Yet, the question arises as to whether patients maintain improved adherence once incentives have stopped. Would incentives have a lasting positive effect on patients' attitudes and behaviour resulting in ongoing improved adherence levels, or would the incentivised behaviour deteriorate again once the incentives are discontinued? Studies in some health-related behaviours such as smoking cessation,^9^ drug abuse^10^ or engagement and retention in HIV treatment and prevention^11^ ^12^ suggest that positive changes in response to financial incentives return to baseline, once the incentives have been removed. Other reports indicate that in the long term, incentivised behaviours may even fall below baseline levels,^13^ ^14^ therefore having a corruptive effect in the long run.

The aim of this study was to assess long-term outcomes of offering financial incentives to improve adherence to LAIs over 2 years after the incentives had been discontinued.

## Methods

The FIAT (Financial Incentives for Adherence to Treatment) trial^5^ was a cluster randomised trial with a 1:1 allocation ratio of mental health teams to the intervention or control condition. It included 141 patients with a diagnosis of schizophrenia, schizoaffective disorder or bipolar disorder, whose adherence to LAIs—calculated as the percentage of LAIs received out of those prescribed over a given period of time—in the 4-month screening period was ≤75%, were recruited from 73 community teams across England and Wales. Written informed consent was obtained from both patients and consultant psychiatrists/team managers. Teams were randomised to either the intervention group in which patients received £15 for each LAI, or the control group which were not offered any incentives. Apart from offering incentives in the intervention group, both groups continued with treatment as usual. The intervention lasted for 12 months. All patients were treated in the National Health Service in England where all treatments, including LAIs, were free of charge for all patients all the time.

Recruitment and randomisation procedures, the sample characteristics and the main findings are reported in detail elsewhere.^5^ ^6^ In brief, the baseline mean adherence of 69% (SD=16%) and 67% (SD=16%) in the intervention and control group, respectively, improved over the 12-month intervention period to 85% (SD=15%) and 71% (SD=22%). The difference between the two groups was statistically significant (adjusted difference in means (β)=11.5%, 95% CI (3.9% to 19.0%), p=0.0003). Patients in the intervention group also had a significantly better subjective quality of life at the end of the 1-year period.

Patients were followed up after the end of the intervention for another 24 months. Outcomes were assessed based on medical records for two separate intervals, that is, the first 6 months and the subsequent 18 months, to distinguish between shorter-term and longer-term outcomes. The assessments for the first 6 months after the end of the intervention were conducted between September 2011 and May 2013, and assessments for the subsequent 18 months between March 2013 and November 2014.

### Primary and secondary outcomes at follow-up

*Primary outcome* was adherence to LAIs, again calculated as the percentage of LAIs received out of those prescribed over the relevant time periods, that is, over 6 months and over an additional 18 months post intervention. *Secondary outcomes* were LAI adherence ≥95%, psychiatric hospital admissions and untoward incidents including police arrest, violent acts and suicide attempts. As in the analysis of the effects during the intervention period, in the calculation, adherence periods spent in hospitals were not considered.

### Statistical analyses

#### Primary outcome

A linear mixed effects regression model with a random effect for the clinical treatment team in which patients received care was used to examine the differences in adherence levels during the 6-month follow-up data. We adjusted for the adherence measure at baseline, MINI International Neuropsychiatric Interview score of team catchment area at randomisation^15^ and the average prescription cycle of LAIs during the baseline period, which depending on the type of medication and specific prescription varied between every week and every 4 weeks.

A simple linear regression model including only a fixed effect for allocation was fitted to the additional follow-up data for the 18 months period between month 7 and month 24. As the intraclass correlation for adherence was negative (−0.05), we did not include a random effect for team. Various sensitivity analyses were conducted: (A) setting adherence of patients with reports of refusing medication to 0%, (B) setting adherence of patients transferred to primary care to 100% and (C) including patients with less than 4 months' adherence data during the 6 month follow-up period.

#### Secondary outcomes

For the initial follow-up period of 6 months and the subsequent 18 months, achieving at least 95% adherence was analysed using mixed effects logistic regression models as described for the primary outcome analysis. This adherence level was chosen to reflect quasi complete adherence. In line with the statistical analyses plan, hospital admissions and adverse events were summarised descriptively at both time intervals due to insufficient power to detect any differences between groups.

## Results

**Figure 1:**
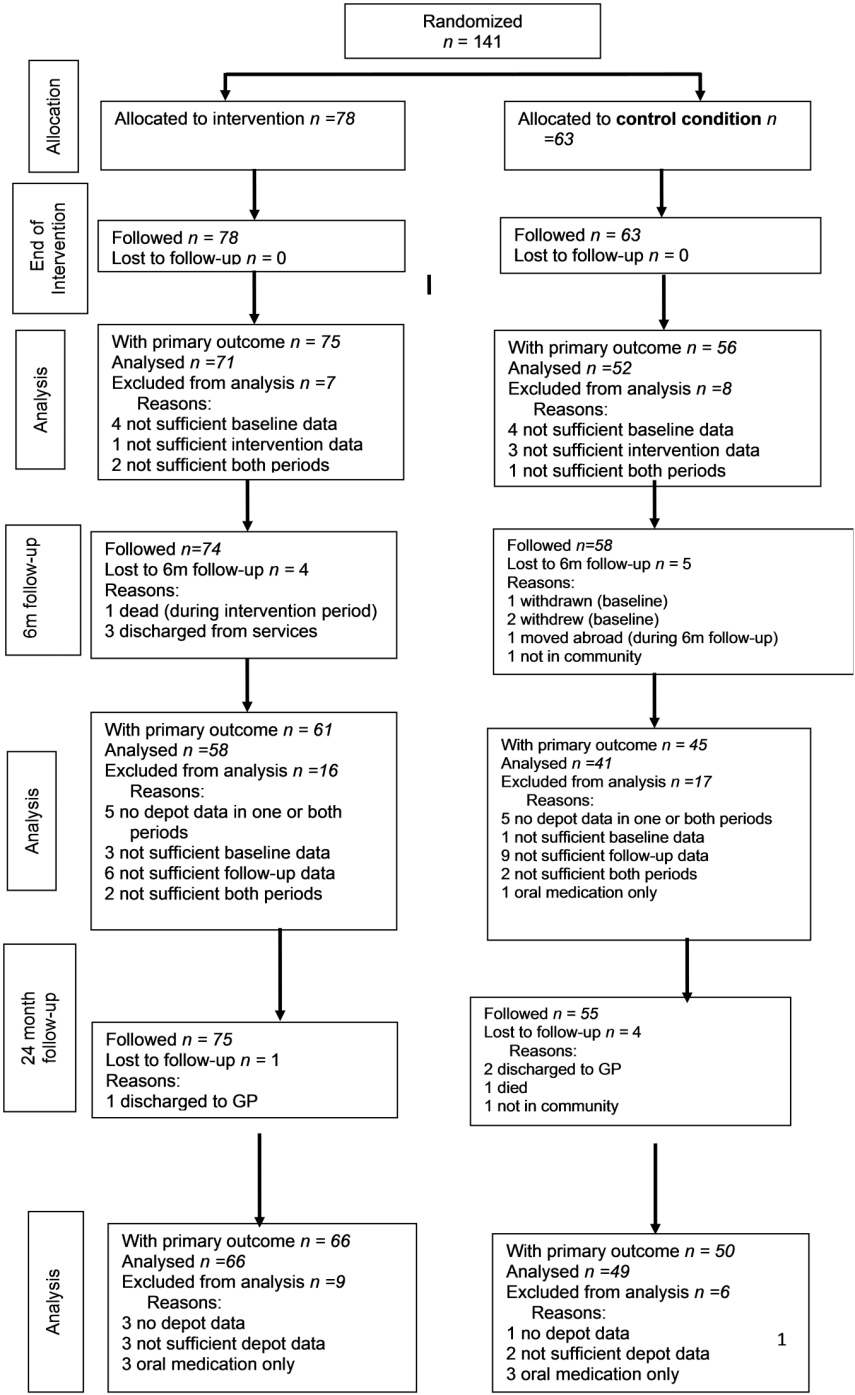


The flow of the participants throughout the duration of the study and socio-demographic and clinical characteristics are presented in the CONSORT diagram (figure 1) and table 1, respectively. Of the 141 randomised patients, 9 were lost to the 6-month follow-up. Of the 132 remaining, the primary outcome could be defined for 106 patients. However, only 99 (58 intervention patients and 41 control patients) had the primary outcome defined at both baseline and 6-month follow-up. At 24 months post intervention, 131 patients remained: additional five patients were lost from the 6-month follow-up, while four patients lost to 6-month follow-up returned to the study (ie, two patients, who had moved away, and two who had been out of the community or discharged to primary care). The primary outcome could be defined for 116 patients (ie, for 66 intervention patients and 50 control patients). This equates to overall follow-up rates of 75.2% after 6 months and 82.3% after 24 months.

**Table 1:** Socio-demographic and clinical characteristics at baseline, end of intervention and 6-month and 24-month follow-ups

	Baseline				End of Intervention				6-month follow-up				24-month follow-up			
	Incentives (N=78)		Control (N=63)		Incentives (N=77)		Control (N=60)		Incentives (N=76)		Control (N=60)		Incentives (N=77)		Control (N=59)	
	Mean or n	SD or %	Mean or n	SD or %	Mean or n	SD or %	Mean or n	SD or %	Mean or n	SD or %	Mean or n	SD or %	Mean or n	SD or %	Mean or n	SD or %
Demographics																
Age (years)	44.4	9.6	42.7	10.2	45.2	9.3	43.3	10.0	45.6	9.3	43.7	10.0	47.2	9.3	45.5	9.9
Male sex	59	76%	46	73%	59	76%	45	76%	58	76%	46	77%	59	77%	45	76%
Ethnicity																
White	49	63%	34	57%	49	63%	33	56%	47	62%	34	57%	48	62%	33	56%
Black	17	22%	14	23%	17	22%	14	24%	17	22%	14	23%	17	22%	14	24%
Asian	5	6%	4	7%	5	6%	4	7%	5	7%	4	7%	5	6%	4	7%
Mixed and other	7	9%	8	13%	7	9%	8	14%	7	9%	8	13%	7	9%	8	14%
Living situation																
Married/co-habiting	8	10%	10	16%	8	10%	7	12%	6	8%	8	13%	12	16%	9	15%
Independent accommodation	53	68%	49	83%	53	68%	50	85%	52	68%	47	81%	48	64%	45	80%
Living alone	41	62%	34	62%	43	55%	35	59%	40	63%	34	64%	44	60%	33	69%
Paid employment (any)	3	4%	1	2%	4	5%	0	0%	4	5%	2	3%	4	5%	1	2%
Receiving benefits	76	99%	58	100%	70	97%	54	98%	70	97%	57	97%	68	96%	48	100%
Diagnosis																
Schizophrenia	61	78%	52	82%	56	81%	45	83%	57	75%	45	75%	56	73%	45	76%
Schizoaffective disorders	9	12%	8	12%	8	12%	5	9%	11	14%	11	18%	11	14%	7	12%
Bipolar affective disorder	6	8%	1	2%	4	6%	3	6%	5	7%	3	5%	5	7%	4	7%
Other psychosis	2	2%	1	2%	1	1%	0	0%	2	3%	0	0%	1	1%	0	0%
Other diagnosis	0	0%	1	2%	0	0%	1	2%	1	1%	1	2%	3	1%	3	5%
Clinical history																
Duration of illness (years)	18.2	8.6	17.3	8.5	19.2	8.6	18.3	8.6	19.9	8.6	18.8	8.5	21.2	8.6	20.5	8.5
Number of psychiatric hospitalisations over assessment period	0.9	2.7	59	0.6	0.7	1.4	0.6	1.4	0.3	0.7	0.2	0.6	0.9	2.3	0.5	1.2

As we have previously shown, less frequent cycles were linked with better adherence.^16^ The changes in treatment cycles throughout the study and associated adherence levels are presented in table 2 and table 3, respectively.

**Table 2:** Treatment cycles during baseline, intervention period and during the two follow-up periods

Depot cycle	Baseline				End of Intervention				1–6-month follow-up				7–24-month follow-up			
	Incentives		Control		Incentives		Control		Incentives		Control		Incentives		Control	
	N	%	N	%	N	%	N	%	N	%	N	%	N	%	N	%
1/52	4	5.6	3	5.5	2	2.7	1	1.8	2	3.3	3	6.7	3	4	1	2
2/52	50	69.4	34	61.8	51	68.0	29	51.8	38	62.3	20	32.8	44	63	24	44
3/52	5	6.9	2	3.6	4	5.3	3	5.4	4	6.6	2	4.5	3	7	1	2
4/52	12	16.7	13	23.6	13	17.3	18	32.1	11	18.0	13	28.9	11	16	20	37
Variable	1	1.4	3	5.5	5	6.7	5	8.9	6	9.8	7	15.6	7	10	8	15
Total	72	100	55	100	75	100	56	100	61	100	45	100	70	100	54	100

**Table 3:** Adherence by treatment cycle at baseline, during the intervention period and during follow-up periods

Treatment cycle	Baseline period (N=123)				Intervention period (N=123)				1–6-month follow-up (N=110)				7–24-month follow-up (N=110)			
	Incentives (N=71)		Control (N=52)		Incentives (N=71)		Control (N=52)		Incentives (N=66)		Control (N=54)		Incentives (N=66)		Control (N=54)	
	N	Mean adherence (%)	N	Mean adherence (%)	N	Mean adherence	N	Mean adherence (%)	N	Mean adherence (%)	N	Mean adherence (%)	N	Mean adherence (%)	N	Mean adherence (%)
1/52	2	54	1	45	2	82	1	49	3	76	3	64	3	78	1	85
2/52	49	67	27	68	49	83	27	74	41	71	23	74	41	67	24	73
3/52	4	76	3	66	4	97	3	44	4	77	2	83	5	74	1	77
4/52	12	76	16	66	12	92	16	73	13	71	20	74	11	66	18	73
5/52	n/a	n/a	n/a	n/a	n/a	n/a	n/a	n/a	1	71	0	n/a	n/a	n/a	n/a	n/a
Variable cycle	4	60	5	62	4	72	5	72	4	59	6	87	6	68	6	75

n/a, not applicable.

The presented descriptive data point to a greater transitioning to less frequent cycles in the control group than in the intervention group throughout the study.

Although the study protocol did not influence whether or not incentives in the intervention group would be continued after the 1-year study period, in practice no patient was offered further incentives by their care teams.

### 

#### Primary outcome

To aid transparency in the change in outcomes throughout the study, table 4 presents the primary outcome at all assessment periods. Mean adherence calculated on all available cases for the 6-month and further 18-month follow-up periods was 71% and 68% in the intervention group, and 78% and 74% in the control group. The difference between the groups was not statistically significant at either time point (adjusted means difference for first 6 months −7.4%, 95% CI −17.0 to 2.1, p=0.175; for following 18 months −5.7, 95% CI −13.1% to 1.7%, p=0.130).

**Table 4:** Primary and secondary outcomes at baseline, end of intervention and 6-month and 24-month follow-ups

		Incentives		Control					
	Period	n* 74†/75‡	Number (%) or means (SD)	n* 58†/56‡	Number (%) or means (SD)	Type of effect estimate	Adjusted effect estimate (intervention vs control)	p Value	ICC
*Primary outcome*									
Adherence (percentage) to depot medication	Baseline	72	69% (16%)	55	67% (16%)				
	12-month intervention	75	85% (15%)	56	71% (22%)	Difference in means¶	11.5% (3.9% to 19.0%)**	0.0003	0.28
	1–6-month follow-up	58	70% (24%)	41	77% (19%)	Difference in means¶	−7.4% (−17.0% to 2.1%)	0.127	0.175
	7–24-month follow-up	66	68% (21%)	50	74% (19%)	Difference in means	−5.7% (−13.1% to 1.7%)	0.130	n/a
*Secondary outcomes*									
Achieving at least 95% adherence vs not	Baseline	72	5 (7%)	55	1 (2%)				
	12-month intervention	75	21 (28%)	56	3 (5%)	OR¶	8.21 (2.00 to 33.67)	0.003	0.04
	1–6-month follow-up	66	5 (8%)	54	9 (17%)	OR¶	0.42 (0.11 to 1.61)	0.205	<0.001
	7–24-month follow-up	66	4 (6%)	50	5 (10%)	OR	0.42 (0.06 to 3.02)	0.392	0.42
At least one psychiatric hospital admission	Baseline	78	14 (19%)	60	10 (17%)				
	12-month intervention	78	15 (19%)	59	14 (24%)				
	1–6-month follow-up	74	15 (20%)	58	8 (14%)				
	7–24-month follow-up	77	24 (31%)	60	10 (17%)				
At least one suicide attempt vs none	Baseline	78	9 (12%)	59	7 (12%)				
	12-month intervention	77	8 (10%)	58	4 (7%)				
	1–6-month follow-up	73	3 (4%)	58	3 (5%)				
	7–24-month follow-up	75	5 (7%)	58	3 (5%)				
At least one violent incident vs none	Baseline	77	15 (20%)	60	10 (17%)				
	12-month intervention	77	10 (13%)	58	7 (12%)				
	1–6-month follow-up	73	4 (6%)	58	3 (5%)				
	7–24-month follow-up	75	11 (15%)	58	7 (12%)				
At least one police arrest vs none	Baseline	77	13 (18%)	60	9 (16%)				
	12-month intervention	77	10 (13%)	60	10 (17%)				
	1–6-month follow-up	73	6 (8%)	58	3 (5%)				
	7–24-month follow-up	75	14 (19%)	58	10 (17%)				

*n is the number of patients in either group with both a baseline and follow-up period for the specified outcome.

†Number of patients during 6-month follow-up. Excludes 9 patients on whom no data were collected during the 6-month follow-up period: 2 who withdrew immediately after randomisation and 1 who was withdrawn as was not being prescribed depot medication, 1 who died during the intervention period, 2 who were discharged during the intervention period, 1 who was discharged during the 6-month follow-up period, 1 who moved abroad during follow-up and 1 who was out of the community for all of the follow-up period.

‡Number of patients during 24-month follow-up. Excludes 4 patients who were lost to follow-up before the start of the 6-month follow-up period. Of the remaining 137 patients, 5 were lost during the 6-month follow-up and a further 5 were lost to follow-up during the final phase of the study. Four patients lost during the 6-month follow-up returned to the study.

¶Each model was adjusted for baseline measure of outcome, MINI International Neuropsychiatric Interview score category (low vs high) and average treatment cycle during baseline, and includes a random effect for team. ICC, Intraclass Correlation Coefficient.

The results of the sensitivity analyses were very similar to the main analyses, that is, all reflected slightly lower adherence in the intervention group with the difference not being statistically significant (table 5).

**Table 5:** Sensitivity analysis of primary outcome at the two follow-up periods

	1–6-month follow-up				7–24-month follow-up			
Analysis population	N	Difference in mean adherence	95% CI	p Value	N	Difference in mean adherence	95% CI	p Value
Main analysis: all participants with ≥4 months' depot data	108	−6.2%	−13.1% to 0.7%	0.078	116	−5.7%*	−13.1% to 1.7%	0.130
All participants as above, setting adherence to 100% for those discharged to GP	130	−5.0%	−14.7% to 4.7%	0.316	119	−6.2%*	−13.6% to 1.1%	0.097
All participants as above but setting adherence to 0% for refusers	112	−4.9%	−14.3% to 4.2%	0.312	124	−6.4%†	−15.0%—2.1%	0.142

*Simple linear regression model including only a fixed effect for intervention versus control. Clustering by team ignorable as the ICC was −0.05.

†Linear mixed effects model including only a fixed effect for intervention versus control. The model-based ICC was p<0.001. GP, General Practitioner.

#### Secondary outcomes

The effect of financial incentives on secondary outcomes through the study is presented in table 5. The percentage of patients achieving at least 95% adherence during the 6-month and further 18-month follow-up was not significantly different between groups (adjusted OR at 6 months=0.42, 95% CI 0.11 to 1.61, p=0.205; and at 24 months=0.42, 95% CI 0.06 to 3.02, p=0.392). No substantial differences were noted in adverse events in either follow-up period. However, there were a higher number of hospital admissions in the intervention group in 6-month and 24-month follow-ups, a difference which, according to our prespecified analysis plan, was shown with only descriptive statistics.

## Discussion

Once financial incentives stop after a year, adherence levels to LAIs return approximately to the levels at the time before the intervention had started. While the positive effect of incentives was consistent for the 1-year intervention period, the return to baseline levels occurs in the first 6 months and remains stable for the 2 years that patients were followed up in this study. Adherence levels in the intervention group were even lower than in the control group, but the differences are small and not statistically significant. The findings suggest that improvements are not sustained, but do not provide any evidence for a negative long-term impact—so called ‘crowding out’—either. No statistically significant differences were found for the secondary outcomes, although descriptively patients in the intervention group showed an increased number of hospital admissions in comparison to the control group in both follow-up periods.

### Strengths and limitations

The study followed patients up over a 2-year period after the 1-year intervention. No patient in the intervention group had been offered further incentives after the research determined 1-year intervention period. One may wonder whether stopping incentives for all patients despite significant improvements in adherence and quality of life of some patients was clinically—and ethically—appropriate. Yet, the fact that no patient continued to be offered incentives simplified the evaluation of long-term effects as there was no variation in the duration of the intervention. The findings for the first 6 months and the subsequent 18 months were similar, suggesting a consistent effect over time.

The follow-up study also has several limitations. The most important is that follow-up data were not complete. While the 1-year outcome data in the original 1-year study had only a small number of dropouts, the dropout rate substantially increased during the 2-year follow-up period. While the overall follow-up rates of 75% and 82% may be regarded as reasonable, missing data may be a particular problem in this type of study. Missing data were due to a number of reasons, such as referral of the patient to primary care or a complete loss of contact with services. These reasons may reflect different scenarios and outcomes. In any case, missing data are unlikely to have occurred at random. It is impossible to assess with certainty whether and, if so, how this selection influenced the results. However, sensitivity analyses with extreme assumptions about the missing data did not alter the findings. Adherence rates were also associated with the prescription cycle which changed differently in the two groups with a tendency towards longer cycles in the control group, and longer cycles were overall associated with better adherence rates. Yet, the changes within each group were inconsistent and the numbers too small for a separate analysis of subgroups with specific patterns of changes. Further limitations are that patients were not interviewed again, so that we did not assess effects and side effects of the medication, and could not explore whether the significant advantages of the intervention group in subjective quality of life were maintained or also lost during the follow-up; and that the study was not powered to establish whether the small adherence differences in favour of the control group were statistically significant or to detect statistically significant differences in rarer secondary outcomes such as rehospitalisations.

### Implications

The findings are consistent with previous literature that suggests that offering financial incentives may improve health-related behaviours for as long as they are provided. This applies to both general^17^ and mental health populations.^18^ ^19^ While a recent systematic review on the effectiveness of financial incentives on health-related behaviours including smoking cessation, diet, reduced alcohol consumption and physical activity suggested that positive effects might last for a short period (ie, 3 months) beyond the provision of incentives,^20^ there is strong evidence suggesting that these benefits are not sustained and that there are no positive long-term effects.^9^ ^10^ ^19^ The findings of our study show that this applies to improved adherence to antipsychotic maintenance medication too.

The FIAT study as a whole has provided clear findings with direct clinical implications. The follow-up study complements the picture of the main trial. The number of patients in the NHS in England who fulfil the inclusion criteria of this study has been estimated to be lower than 1800 at one time.^6^ For these patients, financial incentives improve adherence to antipsychotic depot medication for as long as they are offered, at least for a full year. However, the potential hope that patients with improved adherence may have experienced sufficient benefits to maintain the improved adherence level even when incentives are stopped seems to be unjustified. At least on a group level, there is no evidence for such sustained benefit. On the other hand, there is no evidence either that offering incentives would lead to negative long-term effects once they are stopped. When financial incentives are considered to improve adherence to antipsychotic depot medication, potential positive or negative long-term effects may not be a main criterion.
